# The notable relatedness between ESBL producing *Enterobacteriaceae* isolated from clinical samples and asymptomatic fecal carriers

**DOI:** 10.1186/s12879-023-08746-3

**Published:** 2023-11-08

**Authors:** Shadi Aghamohammad, Fereshteh Shahcheraghi

**Affiliations:** Department of Bacteriology, Pasteur Institute of Iran, Tehran, Iran

**Keywords:** Fecal carriage, Clinical sources, Clonal relatedness, *Enterobacteriaceae*

## Abstract

**Background:**

The investigation of the presence of extended-spectrum beta-lactamase (ESBL) within *Enterobacteriaceae* in both fecal carriers and patients is an essential matter. Furthermore, the assessment of distinct characteristics exhibited by resistant bacteria obtained from fecal carriers and patients, as well as the comparison of these characteristics between the two groups, could provide a deeper understanding of how the resistant isolates can remain concealed within a dormant reservoir and intensify antimicrobial resistance. The aim of the present study was to concentrate on the comparison of the antimicrobial resistance pattern and molecular features between strains obtained from clinical and carrier sources.

**Material and methods:**

A total of 142 clinical samples and 120 rectal swabs were collected from June to October 2016. ESBL screening was performed using the double-disk synergy test. PCR was done for the detection of ESBL genes. Assessment of biofilm formation, virulence factor genes, and MLVA was performed for *K. pneumonae* isolates. Phylogroup typing was performed for *E. coli* isolates.

**Results:**

Of 146 samples, 67.6% were *E. coli,* and 32.4% were *K. pneumoniae.* The rate of *bla*_CTXM-15_ was 89.4%. In *K. pneumoniae* type D, *ompk35* and *fimH* were the highest. All the *K. pneumoniae* isolates were classified into 12 mini clusters and the clinical isolates were characterized into 7 mini clusters. The phylogroup B2 in ESBL-EC was the highest (56.2%).

**Discussion:**

Comparison of molecular characteristics and clonal relatedness between fecal carriers and patients showed noticeable relatedness and similarity which may indicate that ESBL-KP can be colonized with the same profiles in different settings and, therefore, may be widely distributed in both community and hospital settings. Therefore, implementation of control protocols, including surveillance of the fecal carriers, could impressively reduce silent reservoirs without clinical symptoms as well as patient rates.

**Supplementary Information:**

The online version contains supplementary material available at 10.1186/s12879-023-08746-3.

## Background

*E. coli* and *K. pneumoniae* are two common members of the *Enterobacteriaceae* family that cause infections in human including, cystitis and pyelonephritis as urinary tract infections, septicemia,, and meningitis, bacteriemia, respiratory, wound and intra-abdominal infections [1]. In addition to the wide range of infections, the emergence of antibiotic resistance among bacterial pathogens is a worrisome challenge that could affect the healthcare system. Antimicrobial resistance is a silent tsunami that numerous guidelines and recommendations have been reported to combat in modern medicine [2]. Extended-spectrum beta-lactamase (ESBL) production is one of the resistance mechanisms and ESBL-producing *Enterobacteriaceae* (ESBL-PE) could be easily transmitted especially in region with poor hygiene. Antibiotic use is one of the risk factors for the development of resistant bacteria. Studies have reported that various members of antibiotic classes, including ciprofloxacin, mecillinam, and trimethoprim/co-trimoxazole increased the rate of resistant *E. coli* [3]. Also, the presence of co-resistance, e.g. co-resistance to extended-spectrum cephalosporins and fluoroquinolones, may exacerbate the trend of treatment and limit antibiotic choice [4].

Aside from clinical infections, the incidence of fecal carriage has recently gained importance. Rates of ESBL-PE carriers vary among regions, however, the highest rate are found in the western Pacific, eastern Mediterranean, and southeast Asia. The rate of fecal carriers in hospital wards, especially in the intensive care unit (ICU), is considerable and many countries have policiesto screen the carriers at the beginning of admission [5]. In addition to the hospital settings, the rate of fecal carriage in the community is also notable, as ESBL-PE may colonize healthy individuals without clinical symptoms [6]. Several risk factors, including past hospitalization and prior antibiotic use, paly an important role in increasing the likelihood of being a fecal carrier [7]. Socioeconomic status along with foreign travel are other risk factors that may affect the occurrence of fecal carriers [8].

In Iran, a large number of studies have been conducted in recent years to investigate ESBL-PE in clinical infections [9, 10]. Recently, some studies have been conducted looking at the rate of ESBL-PE in fecal carriages [11, 12]. Evaluation of the different characteristics of ESBL-PE strains isolated from clinical samples and fecal carriages could provide important data. Phylogroup typing, for instance, is a PCR-based method that classifies *E. coli* into seven major groups, including A, B1, B2, C, D, E, and F. This classification is important since it could show the potency of virulence of *E. coli* isolates. The determination of the phylogroup types and also typing methods, such as multiple locus variable-number tandem repeat analysis (MLVA), in carrier and clinical samples would be notable as it could reveal the origin of strains. Some of the data from our fecal carriages have been reported, previously [11, 13]. The current study aimed to evaluate the different characteristics of ESBL-PE in clinical samples and also asymptomatic fecal carriers and to compare molecular findings between clinical and carrier samples. To the best of our knowledge, this is the first comparative study between ESBL-PE isolated from clinical samples and fecal carriers, in Iran.

## Methods

### Bacterial isolates

This cross-sectional study was conducted from June to October 2016 in a public hospital in Iran. A total of nonduplicate 142 different clinical samples were collected from 142 hospitalized patients (one sample per patient) admitted to Loghman Hakim Educational Hospital. These specimens included urine, wounds, joints, trachea, and other body fluids. Isolates were identified by standard biochemical tests to identify members of *Enterobacteriaceae*. In addition to evaluating the different molecular characteristics of the clinical isolates, a comparison was also made with the fecal carriages data. The corresponding files of the fecal carriages could be found in the supplementary data. Additionally, previous reports have detailed the quantity and methodology employed in the sampling of fecal carriers [11, 13]. This study was approved by the Ethical Committee of the Pasteur Institute of Iran (IR.PII.REC.1395.44).

### Antimicrobial susceptibility test

Antimicrobial susceptibility testing was performed using the disk diffusion method according to clinical and laboratory standards institute (CLSI) guidelines. The susceptibilities of bacterial isolates against 11 antibiotics ceftazidime (CAZ: 30 mg), CAZ/ clavulanic acid (CAZ/CLA: 30/10 mg), cefotaxime (CTX: 30 mg), CTX/ CLA (30/10 mg), cefepime (CPM: 30 mg), amikacin (AK: 30 mg), gentamicin (GM: 30 mg), ciprofloxacin (CIP: 5 mg), levofloxacin (LVX: 5 mg), ertapenem (ETP: 10 mg), and imipenem (IMP: 10 mg) (MastGroup Ltd., Merseyside, United Kingdom) were tested by agar disk diffusion method. *E. coli* ATCC 25922 was used as a control for the disk diffusion method. Also, the double-disk synergy test (DDST) was used for the phenotypic detection of ESBL producers according to the CLSI guidelines. *K. pneumoniae* ATCC 700603 and *E. coli* ATCC 25922 were used as positive and negative controls in the DDST method, respectively. Also, MDR isolates were classified based on the guideline as having acquired resistance to at least one agent in three or more antimicrobial categories [14].

### Molecular detection of ESBL genes

Total nucleic acid (TNA) extraction was performed using a DNA extraction kit (Bioneer Company, Korea) according to the manufacturer’s instructions. The extracts were stored at -80**°**C until use. PCR assays were carried out for the detection of *bla*_TEM_, *bla*_SHV_, *bla*_CTX-M15_, *bla*_VEB_, and *bla*_PER_ [11].

### Biofilm formation in ESBL-producing *K*. *pneumoniae* (ESBL-KP) isolates using wrinkled colony development

Wrinkled colony development was used to determine the potency of biofilm formation, according to the protocol that was described, previously [15]. Clinical isolates of *K. pneumoniae* were cultured overnight on LB agar, after which a single colony was inoculated in LB broth and agitated at a speed of 180 rpm at a temperature of 37°C until the optical density at 600nm reached 0.2. Subsequently, five micro liters of LB broth containing bacteria were placed on LB agar and incubated at a temperature of 18°C for a duration of 24 h. The morphologies of the wrinkled colonies were then evaluated using a light microscope, and micrographs of the colonies were obtained. The wrinkled colonies were classified into four morphology types (A, B, C, and D), based on the background of the colonies in the presence of light, the state of the bacteria in the molecular matrix, and the thickness of the surrounding colonies.

### Assessment of virulence factors in ESBL-KP isolates

Virulence factors including adhesions (*fimH, mrkD, mrkA, fimA,* and *ECP*), FimH-like-adhesion (*kpn*), mucoviscosity-associated gene A (*magA*), enterobactin (*entB*), outer membrane protein (*ompk35* and *ompk36*), and capsule serotype *K1* and *K2* were evaluated by PCR assay [16, 17].

### Multiple locus variable-number tandem repeat analysis (MLVA) in ESBL-KP isolates

MLVA of ESBL-KP isolates was performed by PCR amplification using six VNTR loci (A, D, E, H, J, and K) with variable numbers of tandem repeats. The PCR conditions and list of primers have been described previously [18]. GelCompar II version 4.0 software (AppliedMaths, Sint-Matens-latem, Belgium) was used for clustering of MLVA types. Clustering was determined by the UPGMA method with a similarity cutoff of 80%. In addition, the diversity indices of the VNTR loci were measured using the Simpson's diversity index. In addition to the clinical isolates, MLVA analysis was also performed to evaluate the clonal relatedness between samples isolated from clinical and fecal carriages [11].

### Phylotyping method in ESBL-producing *E. coli* (ESBL-EC) isolates

Seven phylogroups, including A, B1, B2, C, D, E, and F, were detected using the PCR method described by Clermont et al. [19]. The major target genes, including *chuA*, *yjaA*, *TspE4*.*C2*, and *arpA*, were amplified by PCR. To characterize phylogroups C and E, additional allele-specific PCR primers were used [13].

### Statistical analysis

Statistical analyses were performed using SPSS software (version 25; SPSS, Inc., Chicago, IL, USA). The Chisquare test was applied to analyze categorical data. A *p*-value < 0.05 in all experiments was considered statistically significant. MLVA excel files of isolates were analyzed by PHYLOViZ 2.0 software.

## Results

### Detection of bacterial isolates

From a total of 146 clinical samples, 96 (67.6%) were *E. coli* and 46 (32.4%) were identified as *K. pneumoniae.* Urine (66/96, 68.7%) and trachea (21/46, 45.6%) were the most two sources that *E. coli* and *K. pneumoniae* were isolated. *K. pneumoniae* was also isolated from urine (8/46, 17.4%), joint (6/46, 13%), wound (6/46, 13%), surgery site (3/46, 6.5%), blood (1/46, 2.2%), and ascites (1/46, 2.2%). The rates of *E. coli* isolates in trachea, blood, surgery site, and abscess were 28.3% (13/96), 10.4% (10/96), 5.2% (5/96), and 2.1% (2/96), respectively. Also, *E. coli* was more isolated from outpatients and *K. pneumoniae* had a higher rate in ICU. All molecular data of ESBL-KP and ESBL-EC could be seen in Tables 1 and 2. The previously published results about 120 rectal swabs (including 22 ESBL-KP and 72 ESBL-EC) isolated from fecal carriages, could be found in the supplementary files. The clinical, phenotypic, and genotypic characteristics of the 22 ESBL-KP isolated from fecal carriage could be found in Table S1. The antimicrobial resistance profile, phylogroup typing, and ESBL genes distribution of 72 ESBL-EC isolated from fecal carriage could be seen in Table S2.

**Table 1:** Clinical, Phenotypic, and Genotypic Characteristics of ESBL-KP Isolated from Clinical Samples

NO	Source	Ward	Non susceptible profile	ESBL genes	Biofilm	Virulence factor genes
K1	U/C	Internal	CAZ,CTX,CPM,AK,GM	*bla*_*CTX*_*,bla*_*SHV*_	A	*fimH,mrkD,mrkA,fimA, entB,ompk35,ompk36,K2*
K2	U/C	Internal	CAZ,CTX,CPM, CIP,LVX,ETP,IMP	*bla*_*CTX*_*,bla*_*TEM,*_*bla*_*SHV*_	A	*fimH,mrkD,mrkA,ecp,KPN,entB,ompk35,ompk36,K2*
K3	Wound	OP	CAZ,CTX,CPM, CIP,LVX,ETP	*bla*_*CTX*_*,bla*_*TEM,*_*bla*_*SHV*_	B	*fimH,mrkD,mrkA,ecp,KPN,entB,ompk35,ompk36*
K4	surgery	surgery	CAZ,CTX,CPM, CIP,LVX,ETP	*bla*_*CTX*_*,bla*_*TEM,*_*bla*_*SHV*_	A	*fimH,mrkD, ecp,KPN, entB,ompk35,ompk36*
K8	U/C	emergency	CAZ,CTX,CPM, CIP,LVX,ETP,IMP	*bla*_*SHV*_	C	*fimH,mrkD,mrkA,ecp,KPN,entB,ompk35,ompk36*
K9	T/C	ICU	CAZ,CTX,CPM, CIP,LVX,ETP,IMP	*bla*_*CTX*_*,bla*_*TEM,*_*bla*_*SHV*_	C	*fimH,mrkD,mrkA, KPN, entB,ompk35,ompk36*
K10	T/C	ICU	CAZ,CTX,CPM, CIP,LVX,ETP,IMP	*bla*_*CTX*_*,bla*_*SHV*_	A	*fimH,mrkD,mrkA,,ecp,KPN, entB,ompk35,ompk36*
K11	T/C	Pediatric	CAZ,CTX,CPM, CIP,LVX	*bla*_*CTX*_*,bla*_*TEM,*_*bla*_*SHV*_	D	*fimH,mrkD,mrkA,fimA, ecp, entB,ompk35,ompk36*
K12	B/C	ICU	CAZ,CTX,CPM, CIP,LVX,ETP	*bla*_*CTX*_*,bla*_*TEM,*_*bla*_*SHV*_	D	*fimH,mrkD,mrkA, ecp,KPN, entB,ompk35,ompk36,K2*
K14	Wound	emergency	CAZ,CTX,CPM, CIP,LVX,ETP,IMP	*bla*_*CTX*_*,bla*_*SHV*_	D	*fimH,mrkD,mrkA,fimA, KPN, entB,ompk35,ompk36,K2*
K15	surgery	surgery	CAZ,CTX,CPM,AK, CIP,LVX,ETP,IMP	*bla*_*CTX*_*,bla*_*SHV*_	C	*fimH,mrkD, fimA, ecp, entB,ompk35,ompk36,K2*
K16	T/C	ICU	CAZ,CTX,CPM, CIP,LVX,ETP,IMP	*bla*_*CTX*_*,bla*_*TEM,*_*bla*_*SHV*_	D	*fimH,mrkD,mrkA, ecp,KPN, entB,ompk35,ompk36,K2*
K17	U/C	emergency	CAZ,CTX,CPM,AK, CIP,LVX,ETP,IMP	*bla*_*CTX*_*,bla*_*TEM,*_*bla*_*SHV*_	B	*fimH,mrkD,mrkA, ecp,KPN, entB,ompk35,ompk36*
K18	T/C	ICU	CAZ,CTX,CPM,GM, CIP,LVX,ETP,IMP	*bla*_*CTX*_*,bla*_*TEM,*_*bla*_*SHV*_	D	*fimH,mrkD,mrkA, KPN, entB,ompk35,ompk36,K2*
K19	Wound	OP	CAZ,CTX,CPM,GM, CIP,LVX	*bla*_*CTX*_*,bla*_*TEM,*_	C	*fimH,mrkD, entB,K2*
K20	U/C	ICU	CAZ,CTX,CPM, CIP,LVX,ETP,IMP	*bla*_*CTX*_*,bla*_*TEM,*_*bla*_*SHV*_	D	*fimH,mrkD,mrkA, ecp,KPN, entB,ompk35,ompk36,K2*
K21	Joint	emergency	CAZ,CTX,CPM	*bla*_*CTX*_*,bla*_*TEM,*_	D	*fimH,mrkD,mrkA,fimA, KPN, entB,ompk35,ompk36*
K22	T/C	ICU	CAZ,CTX,CPM, CIP,LVX,ETP,IMP	*bla*_*CTX*_	B	*fimH,mrkD,mrkA,fimA, KPN, entB,ompk35,ompk36*
K23	T/C	ICU	CAZ,CTX,CPM,AK, CIP,LVX,ETP,IMP	*bla*_*CTX*_*,bla*_*TEM,*_*bla*_*SHV*_	D	*fimH,mrkD, ecp,KPN, entB,ompk35,ompk36*
K24	Ascites	emergency	CAZ,CTX,CPM, CIP,LVX	*bla*_*CTX*_*,bla*_*TEM,*_*bla*_*SHV*_	D	*fimH,mrkD,mrkA, ecp,KPN, entB,ompk35,ompk36,K2*
K25	T/C	ICU	CAZ,CTX,CPM	*bla*_*CTX*_*,bla*_*TEM,*_*bla*_*SHV*_	D	*fimH,mrkD,mrkA,fimA, ecp,KPN, entB,ompk35,ompk36*
K26	T/C	Internal	CAZ,CTX,CPM, CIP	*bla*_*CTX*_*,bla*_*TEM,*_*bla*_*SHV*_	D	*fimH,mrkA, fimA, KPN, ompk35,ompk36*
K27	wound	surgery	CAZ,CTX,CPM,GM,ETP	*bla*_*CTX*_*,bla*_*SHV*_	C	*fimH,mrkD, ecp,KPN, entB,ompk35,ompk36*
K28	Joint	Internal	CAZ,CTX,CPM, CIP,LVX,ETP,IMP	*bla*_*CTX*_*,bla*_*TEM,*_*bla*_*SHV*_	D	*fimH,mrkD, ecp,KPN, entB,ompk35,ompk36,K2*
K29	T/C	ICU	CAZ,CTX,CPM,AK, CIP,LVX,ETP,IMP	*bla*_*CTX*_*,bla*_*TEM,*_*bla*_*SHV*_	D	*fimH,mrkD,mrkA,fimA, ecp,KPN, entB,ompk35,ompk36*
K30	T/C	ICU	CAZ,CTX,CPM, CIP,LVX,ETP,IMP	*bla*_*CTX*_*,*_*,*_*bla*_*SHV*_	C	*fimH,mrkD,mrkA, ecp,KPN, entB,ompk35,ompk36,K2*
K32	T/C	ICU	CAZ,CTX,CPM,GM, CIP,LVX, ETP,IMP	*bla*_*CTX*_*,bla*_*TEM,*_	D	*fimH,mrkD, KPN, entB,ompk35,ompk36*
K33	T/C	ICU	CAZ,CTX,CPM,GM, CIP,LVX, ETP,IMP	*bla*_*CTX*_*,*	D	*fimH,mrkD,mrkA, KPN, entB,ompk35,ompk36*
K34	Joint	emergency	CAZ,CTX,CPM, CIP	*bla*_*CTX*_*,bla*_*TEM,*_*bla*_*SHV*_	D	*fimH,,fimA, KPN, entB,ompk35,ompk36*
K35	T/C	ICU	CAZ,CTX,CPM,GM, CIP,LVX, ETP	*bla*_*CTX*_*,bla*_*TEM,*_*bla*_*SHV*_	D	*fimH,mrkD,mrkA,, ecp,KPN, entB,ompk35,ompk36*
K36	Joint	Internal	CAZ,CTX,CPM,GM, CIP	*bla*_*CTX*_*,bla*_*TEM,*_*bla*_*SHV*_	C	*fimH,,fimA, ecp,KPN, entB,ompk35,ompk36*
K37	T/C	ICU	CAZ,CTX,CPM, CIP,LVX,ETP,IMP	*bla*_*CTX*_*,bla*_*TEM,*_*bla*_*SHV*_	B	*fimH,mrkD,mrkA, ecp,KPN, entB,ompk35,ompk36*
K38	wound	emergency	CAZ,CTX,CPM, CIP,LVX,ETP,IMP	*bla*_*CTX*_*,bla*_*TEM,*_*bla*_*SHV*_	D	*fimH,mrkD,mrkA, KPN, entB,ompk35,ompk36*
K40	T/C	Internal	CAZ,CTX,CPM, CIP,LVX,ETP,IMP	*bla*_*CTX*_*,bla*_*TEM,*_*bla*_*SHV*_	D	*fimH,mrkD,mrkA, ecp,KPN,, entB,ompk35,ompk36*
K41	T/C	ICU	CAZ,CTX,CPM,AK, CIP,LVX	*bla*_*CTX*_*,bla*_*TEM,*_	D	*fimH,mrkD,mrkA, ecp,KPN,, entB,ompk35,ompk36*
K42	Joint	emergency	CAZ,CTX,CPM,GM, CIP,LVX, ETP,IMP	*bla*_*CTX*_*,bla*_*SHV*_	C	*fimH,mrkD,mrkA,fimA, ecp,KPN, entB,ompk35,ompk36*
K43	wound	surgery	CAZ,CTX,CPM,GM, CIP,LVX, ETP,IMP	*bla*_*CTX*_*,bla*_*TEM,*_*bla*_*SHV*_	D	*fimH,mrkD,mrkA, ecp,KPN, entB,ompk35,ompk36*
K44	T/C	ICU	CAZ,CTX,CPM,GM, CIP,LVX, ETP,IMP	*bla*_*CTX*_*,bla*_*TEM,*_*bla*_*SHV*_	C	*fimH,mrkD,mrkA, ecp,KPN, entB,ompk35,ompk36*
K45	surgery	surgery	CAZ,CTX,CPM,GM, CIP,LVX, ETP,IMP	*bla*_*CTX*_*,bla*_*SHV*_	C	*fimH,mrkD,mrkA,fimA, ecp,KPN, entB,ompk35,ompk36*
K46	T/C	ICU	CAZ,CTX,CPM,GM, CIP,LVX, ETP	*bla*_*CTX*_*,bla*_*TEM,*_*bla*_*SHV*_	D	*fimH,mrkD,mrkA, ecp,KPN,, entB,ompk35,ompk36*
K53	T/C	ICU	CAZ,CTX,CPM, CIP,LVX	*bla*_*CTX*_*,bla*_*TEM,*_*bla*_*SHV*_	C	*fimH,mrkD,mrkA, ecp,KPN, entB,ompk35,ompk36*
K54	U/C	Pediatric	CAZ,CTX,CPM,GM, CIP,LVX	*bla*_*CTX*_*,bla*_*TEM,*_*bla*_*SHV*_	D	*fimH,mrkD,mrkA,fimA, ecp,, entB,ompk35,ompk36*
K55	Joint	emergency	CAZ,CTX,CPM,AK,GM, CIP,LVX	*bla*_*CTX*_*,bla*_*TEM,*_*bla*_*SHV*_	C	*fimH,mrkD,mrkA, KPN, entB,ompk35,ompk36*
K56	U/C	emergency	CAZ,CTX,CPM, CIP,LVX	*bla*_*TEM,*_*bla*_*SHV*_	C	*fimH,mrkD,mrkA, ecp,KPN, entB,ompk35,ompk36*
K57	U/C	OP	CAZ,CTX,CPM, CIP,LVX,ETP,IMP	*bla*_*CTX,*_*bla*_*SHV*_	C	*fimH,mrkD,mrkA, ecp,KPN, entB,ompk35,ompk36*
K61	T/C	ICU	CAZ,CTX,CPM,AK, GM,CIP,LVX,ETP,IMP	*bla*_*CTX*_*,bla*_*TEM,*_*bla*_*SHV*_*,*	D	*fimH,mrkD,mrkA, KPN, entB,ompk35,ompk36*

*ICU* Intensive care unit, *OP* Outpatient, *UC* Urine culture, *BC* Blood culture, *T/C* Treacheal culture, CAZ, ceftazidime, *CTX* Cefotaxime, *CPM* Cefepime, *AK* Amikacin, *GM* Gentamicin, *CIP* Ciprofloxacin, *LVX* Levofloxacin, *ETP* Ertapenem, *IMP* Imipenem

**Table 2:** Antimicrobial resistance profile, Phylogroup typing, and ESBL genes distribution of 96 ESBL-EC isolated from clinical samples in Iran

NO	Source	Ward	Non susceptible profile	ESBL genes	Phylogroup type
E73	B/C	Internal	CAZ,CTX,CPM,GM	*bla*_CTX_*,bla*_TEM_	B2
E74	U/C	Pediatric	CAZ,CTX,CPM,CIP,LVX	*bla*_CTX_	B2
E75	U/C	Pediatric	CAZ,CTX,CPM,CIP,LVX	*bla*_CTX_*,bla*_TEM_	B2
E76	U/C	ICU	CAZ,CTX,CPM,CIP,LVX,ETP	*bla*_CTX_	B2
E77	Abscess	ICU	CAZ,CTX,CPM,CIP,LVX	*bla*_CTX_*,bla*_TEM_	B2
E78	Surgery	Internal	CAZ,CTX,CPM,CIP,LVX	*bla*_CTX_	B2
E79	U/C	Skin	CAZ,CTX,CPM,GM,CIP,LVX	*bla*_CTX_	B2
E80	U/C	OP	CAZ,CTX,CPM,CIP,LVX,ETP	*bla*_SHV_	B2
E81	U/C	Pediatric	CAZ,CTX,CPM,CIP,LVX	*bla*_TEM_	B2
E82	U/C	Internal	CAZ,CTX,CPM	*bla*_CTX_*,bla*_TEM_	B2
E83	U/C	OP	CAZ,CTX,CPM,CIP,LVX	*bla*_CTX_*,bla*_TEM_	B2
E84	B/C	OP	CAZ,CTX,CPM,CIP,LVX	*bla*_CTX_	B2
E85	U/C	OP	CAZ,CTX,CPM,CIP,LVX	*bla*_CTX_	B2
E86	U/C	OP	CAZ,CTX,CPM,CIP,LVX	*bla*_SHV_	B2
E87	T/C	ICU	CAZ,CTX,CPM,GM,CIP,LVX	*bla*_CTX_*,bla*_TEM_	B2
E88	U/C	OP	CAZ,CTX,CPM	*bla*_CTX_*,bla*_TEM_	B2
E89	B/C	Emergency	CAZ,CTX,CPM,CIP,LVX	*bla*_CTX_*,bla*_TEM_	B2
E90	U/C	Internal	CAZ,CTX,CPM,CIP,LVX	*bla*_CTX_	B2
E91	U/C	Emergency	CTX,CPM	*bla*_CTX_	B2
E92	T/C	ICU	CAZ,CTX,CPM,CIP,LVX	*bla*_TEM_	B2
E93	U/C	OP	CAZ,CTX,CPM	*bla*_CTX_*,bla*_TEM_	B2
E94	U/C	Emergency	CAZ,CTX,CPM,CIP,LVX	*bla*_CTX_	B2
E95	U/C	Emergency	CAZ,CTX,CPM,CIP,LVX	*bla*_CTX_	B2
E96	U/C	OP	CAZ,CTX,CPM	*bla*_CTX_	B2
E97	U/C	OP	CAZ,CTX,CPM,CIP,LVX	*bla*_CTX_*,bla*_TEM_	B2
E98	U/C	Pediatric	CAZ,CTX,CPM	*bla*_CTX_	B2
E99	T/C	ICU	CAZ,CTX,CPM,GM,CIP,LVX	*bla*_TEM_	B2
E100	U/C	OP	CAZ,CTX,CPM,GM,CIP,LVX	*bla*_CTX_*,bla*_TEM_	B2
E101	U/C	Emergency	CAZ,CTX,CPM,CIP,LVX	*bla*_CTX_	B2
E102	U/C	ICU	CAZ,CTX,CPM,GM,CIP,LVX	*bla*_CTX_*,bla*_TEM_	B2
E103	U/C	OP	CAZ,CTX,CPM,CIP,LVX	*bla*_CTX_	B2
E104	U/C	Skin	CAZ,CTX,CPM,CIP,LVX	*bla*_CTX_	B2
E105	B/C	Emergency	CAZ,CTX,CPM,GM,CIP,LVX	*bla*_CTX_	B2
E106	U/C	Emergency	CAZ,CTX,CPM,CIP,LVX	*bla*_CTX_*,bla*_TEM_	B2
E107	U/C	OP	CAZ,CTX,CPM,CIP,LVX	*bla*_CTX_	B2
E108	U/C	OP	CAZ,CTX,CPM	*bla*_CTX_	B2
E109	B/C	Emergency	CAZ,CTX,CPM,CIP,LVX	*bla*_CTX_	B2
E110	U/C	OP	CAZ,CTX,CPM	*bla*_CTX_	B2
E111	U/C	Internal	CAZ,CTX,CPM,CIP,LVX	*bla*_CTX_*,bla*_TEM_	B2
E112	U/C	OP	CAZ,CTX,CPM,AK,CIP,LVX	*bla*_CTX_	B2
E113	U/C	OP	CAZ,CTX,CPM	*bla*_CTX_	B2
E114	U/C	OP	CAZ,CTX,CPM,GM,CIP,LVX	*bla*_CTX_*,*	B2
E115	T/C	ICU	CAZ,CTX,CPM,CIP,LVX	*bla*_CTX_*,bla*_TEM_	B2
E116	Surgery	Internal	CAZ,CTX,CPM,GM,CIP,LVX	*bla*_CTX_	B2
E117	U/C	Internal	CAZ,CTX,CPM,CIP,LVX	*bla*_TEM_	B2
E118	U/C	ICU	CAZ,CTX,CPM,CIP,LVX	*bla*_TEM,_*,bla*_SHV_	B2
E119	U/C	OP	CAZ,CTX,CPM	*bla*_CTX_*,bla*_TEM_	B2
E120	T/C	ICU	CTX,IMP	*bla*_TEM_	B2
E121	U/C	Emergency	CAZ,CTX,CPM,CIP,LVX	*bla*_CTX_	B2
E122	U/C	ICU	CTX	*bla*_TEM_	B2
E123	B/C	Pediatric	CAZ,CTX,CPM,CIP	*bla*_TEM_	B2
E124	U/C	ICU	CAZ,CTX,CPM	*bla*_TEM_	B2
E125	T/C	Internal	CAZ,CTX,CPM	*bla*_CTX_	B2
E126	T/C	Pediatric	CAZ,CTX,CPM	*bla*_CTX_	B2
E127	Abscess	Emergency	CTX,CIP,LVX	*bla*_CTX_*,bla*_TEM_	E
E128	U/C	Emergency	CAZ,CTX	*bla*_CTX_	E
E129	T/C	ICU	CAZ,CTX,CPM,GM,CIP,LVX	*bla*_CTX_*,bla*_TEM_	E
E130	U/C	Surgery	CTX	*bla*_CTX_	E
E131	Surgery	Surgery	CAZ,CTX,CPM,CIP,LVX	*bla*_CTX_	E
E132	B/C	Emergency	CAZ,CTX,CPM,CIP,LVX	*bla*_CTX_	E
E133	U/C	Internal	CAZ,CTX,CPM	*bla*_CTX_	E
E134	B/C	Skin	CAZ,CTX,CPM,CIP,LVX	*bla*_CTX_	E
E135	T/C	Emergency	CAZ,CTX,CPM	*bla*_CTX_	E
E136	U/C	OP	CTX,CIP,LVX	*bla*_CTX_	E
E137	U/C	Emergency	CAZ,CTX,CPM,CIP	*bla*_CTX_*,bla*_TEM_	E
E138	U/C	Pediatric	CAZ,CTX,CPM,CIP,LVX	*bla*_CTX_	A
E139	U/C	Emergency	CTX,CIP,LVX	*bla*_CTX_*,bla*_TEM_	A
E140	U/C	Emergency	CAZ,CTX,CPM,CIP,LVX	*bla*_CTX_*,bla*_TEM_	A
E141	U/C	Internal	CAZ,CTX,CPM,CIP,LVX,ETP,IMP	*bla*_CTX_	A
E142	U/C	Emergency	CAZ,CTX,CPM,CIP	*bla*_CTX_	A
E143	Surgery	Surgery	CAZ,CTX,CPM	*bla*_CTX_	A
E144	U/C	Emergency	CAZ,CTX,CPM	*bla*_CTX_	D
E145	U/C	OP	CTX	*bla*_CTX_*,bla*_TEM_	D
E146	T/C	ICU	CTX,CIP,LVX	*bla*_CTX_*,bla*_TEM_	D
E147	U/C	ICU	CAZ,CTX,CPM,CIP,LVX	*bla*_CTX_	F
E148	Surgery	Surgery	CTX,CIP,LVX	*bla*_TEM_	F
E149	T/C	Emergency	CAZ,CTX,CPM	*bla*_CTX_	F
E150	B/C	OP	CAZ,CTX,CPM,CIP,LVX,ETP,IMP	*bla*_CTX_	C
E151	U/C	Emergency	CAZ,CTX,CPM,GM,CIP,LVX	*bla*_CTX_	C
E152	U/C	OP	CTX	*bla*_CTX_	B1
E153	U/C	OP	CAZ,CTX,CPM	*bla*_CTX_	UT
E154	U/C	OP	CAZ,CTX,CPM,CIP,LVX	*bla*_CTX_	UT
E155	U/C	OP	CAZ,CTX,CPM,CIP,AK,LVX,ETP,IMP	*bla*_CTX_*,bla*_TEM,_*bla*_SHV_	UT
E156	B/C	Emergency	CAZ,CTX,CPM	*bla*_CTX,_ *bla*_SHV_	UT
E157	T/C	Emergency	CAZ,CTX,CPM,GM,CIP,LVX	*bla*_CTX_	UT
E158	U/C	Pediatric	CAZ,CTX,CPM,CIP,LVX,ETP	*bla*_CTX_	UT
E159	U/C	ICU	CAZ,CTX,CPM,CIP,LVX	*bla*_CTX_	UT
E160	U/C	OP	CAZ,CTX,CPM,CIP,LVX	*bla*_CTX_	UT
E161	U/C	Emergency	CAZ,CTX,CPM,CIP,LVX	*bla*_CTX,_ *bla*_SHV_	UT
E162	U/C	Surgery	CAZ,CTX,CPM,CIP,LVX	*bla*_CTX_*,bla*_TEM_	UT
E163	U/C	OP	CAZ,CTX,CPM	*bla*_CTX_*,bla*_TEM_	UT
E164	T/C	Skin	CAZ,CTX,CPM,CIP,LVX,IMP	*bla*_CTX,_ *bla*_SHV_	UT
E165	U/C	Internal	CAZ,CTX,CPM,CIP,LVX	*bla*_CTX_	UT
E166	U/C	Internal	CAZ,CTX,CPM,CIP,LVX,ETP,IMP	*bla*_CTX_*,bla*_TEM,,_ *bla*_SHV_	UT
E167	U/C	Emergency	CAZ,CTX,CPM,GM,CIP,LVX	*bla*_CTX_*,bla*_TEM_	UT
E168	U/C	ICU	CAZ,CTX,CPM,GM,CIP, ETP,IMP	*bla*_TEM_	UT

*ICU* Intensive care unit, *OP* Outpatient, *UC* Urine culture, *BC* Blood culture, *T/C* Treacheal culture, *CAZ* Ceftazidime, *CTX* Cefotaxime, *CPM* Cefepime, *AK* Amikacin, *GM* Gentamicin, *CIP* Ciprofloxacin, *LVX* Levofloxacin, *ETP* Ertapenem, *IMP* Imipenem, *UT* Untypable

### Antimicrobial susceptibility pattern

DDST showed that all clinical isolates had an ESBL-positive phenotype. The disk diffusion method showed that the highest resistance was seen against cefotaxime (142/142, 100%), ceftazidime (131/142, 91%), and cefepime (129/142, 90%). The rates of resistance to ciprofloxacin and levofloxacin were 76% (110/142) and 71% (102/142), respectively. Resistance to other antimicrobial agents was as follows: ertapenem, 28% (40/142); imipenem, 22% (32/142); gentamicin, 22% (32/142); and amikacin, 7% (10/142). The rates of multidrug resistance (MDR) in ESBL-KP and ESBL-EC isolates were 82.6% (38/46) and 22.9% (22/96), respectively.

### Molecular detection of ESBL

The rates of *bla*_CTX-M15_, *bla*_SHV_ and *bla*_TEM_ genes were 89.4% (127/142), 51.4% (73/142), and 33.8% (48/142) of isolates, respectively. None of the isolates had *bla*_VEB_ and *bla*_PER_ genes. The presence of three ESBL genes simultaneously was significantly higher in ESBL-KP in comparison to ESBL-EC (*P* < 0.05). 63% (29/46) of ESBL-KP harbored three ESBL genes, while only in 2% (2/96) of ESBL-EC the three ESBL genes detected.

### Biofilm formation in ESBL-KP isolates

According to the wrinkled colony development four types, including A, B, C, and D. The rates of types A, B, C, and D were 8.6% (4/46), 8.6% (4/46), 30.4% (3/22), and 52.1% (24/46) of isolates, respectively. The rates of biofilm types in ESBL-KP isolated from fecal carriages could be seen in Figure S1.

### Detection of virulence factor genes in ESBL-KP

*Ompk35* and *fimH* were detected in all ESBL-KP isolates. The rates of other virulence genes were as follows: *ompk 36* 97.8% (45/46), *entB* 97.8% (45/46), *mrkD* 93.4% (43/46), *kpn* 91.3% (42/46), *mrkA* 80.4% (37/46), *ecp* 69.5% (32/46), *fimA* 30.4% (14/46), and *K2* 26% (12/46), respectively. None of the isolates had *K1* and *mag* genes.

### MLVA results in ESBL-KP

**Fig. 1:**
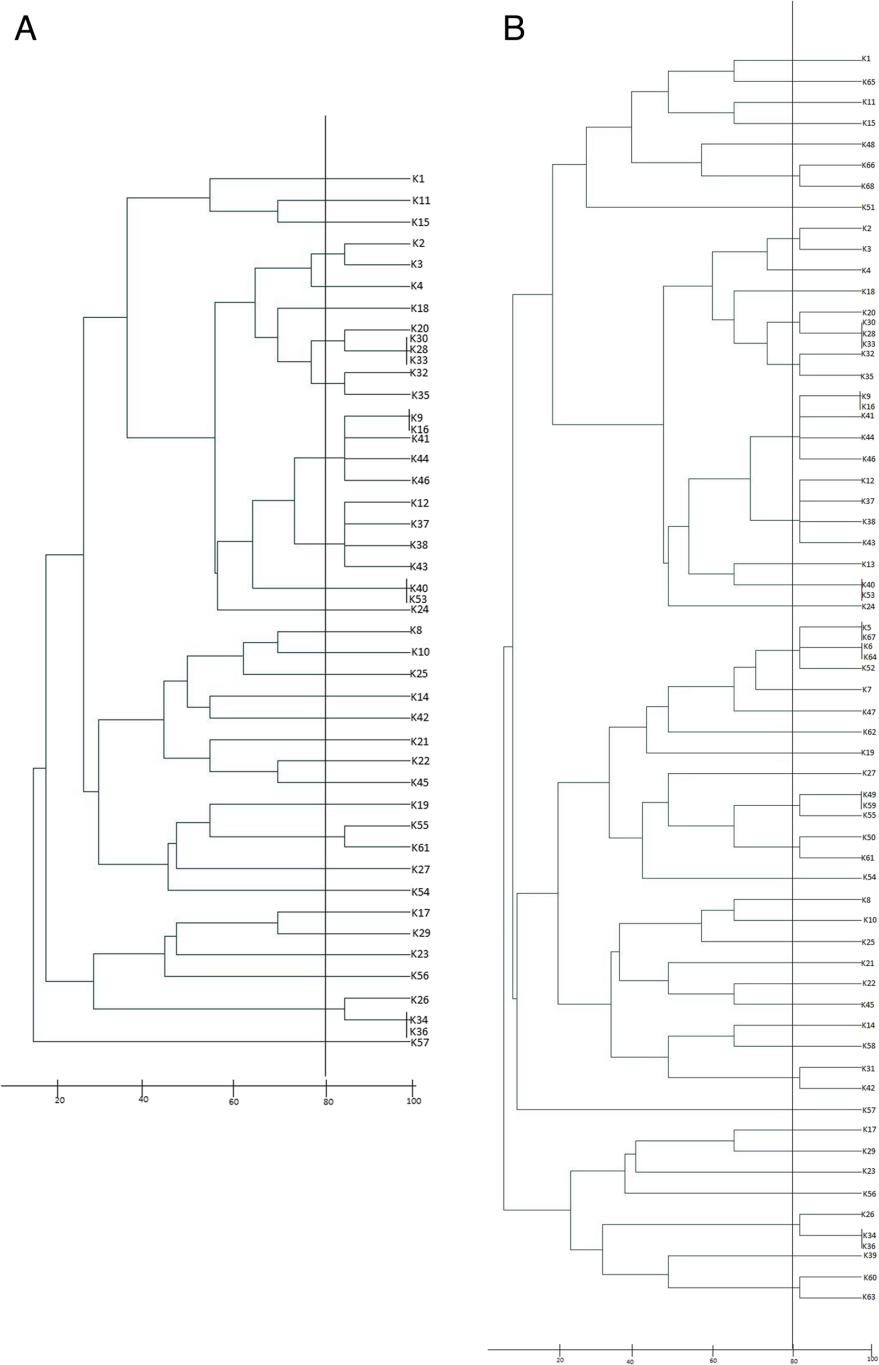
**A** Dendrogram based on MLVA of 46 ESBL-KP in clinical sources, **B** Dendrogram based on MLVA of 68 ESBL-KP strains isolated from clinical sources and fecal carriages

Analysis of six VNTR loci profiles in the ESBL-KP isolated from clinical isolates showed that the lowest diversity index was seen in VNTR_K (0.630) and the highest diversity index was found for VNTR_E (0.885). Based on a cutoff of 80% similarity, the isolates were characterized into 7 mini clusters and 20 singletons (Fig. 1A). Some of the ESBL-KP isolates isolated from different wards of the hospital had exactly the same MLVA profiles (e.g. K9 and K16). Also, analysis of VNTR loci profiles in all the ESBL-KP isolates (isolated from clinical and fecal carriages) demonstrated that the lowest diversity index was seen in VNTR_K (0.645) and the highest diversity index was found for VNTR_E (0.898). At 80% similarity, isolates were divided into 12 mini clusters and 31 singletons (Fig. 1B). The results of MLVA of 22 ESBL-KP isolated from fecal carriage in could be seen in dendrogram in Figure S2.

### Detection of phylogroups in ESBL-EC isolates

The percentage of phylogroup A, B1, B2, C, D, E and F were 6.2% (6/96), 1% (1/96), 56.2% (54/96), 2.1% (2/96), 3.1% (3/96), 11.4% (11/96), and 3.1% (3/96) respectively. Of all isolates, 16.6% (16/96) were classified as untypable. The rate of putative virulent ESBL-EC isolates was 62.5% (60/96) while, the commensal phylogroups were detected in 20.8% (20/96) of isolates.

## Discussion

Several studies evaluated different molecular characteristics at ESBL-PE clinical isolates [20, 21]. The evaluation of ESBL-PE isolated from fecal carriages has also recently gained importance [22]. Evaluation of fecal carriage could be important in different aspects as carriage could lead to infection of the host, transfer of resistance genes to other bacterial species, and spread to others, specifically family members [23]. Comparison of molecular characteristics between clinical isolates and strains isolated from carriers, particularly clonal relatedness, showing similarity of source of dissemination, and phylogroup typing, indicating the tendency to cause disease, may confirm this hypothesis that fecal carriers could further evolve into patients. To the best of our knowledge, no analysis or comparison has been performed between ESBL-PE strains isolated from clinical and fecal carriers during the same time period.

In the present study, the rate of putative virulent ESBL-EC isolates (62.5%) was higher than that of commensal phylogroups (20.8%). Similar results were obtained for fecal carriers. The rate of putative virulent ESBL-EC isolates among fecal carriers was 47.2%, higher than among commensal phylogroups (30.5%). The rate of MDR strains at ESBL-EC isolated from clinical samples (22.9%) was nearly twice the MDR rate for fecal carriages (12.5%). (See also Table S2 in supplementary files). The results showed thatthe rate of antimicrobial resistance and putative virulent groups was higher in clinical isolates as expected. However, the presence of virulent phylogroups, especially phylogroup B2 which is thought to be associated with clinical infections like UTI and also Crohn’s disease, with multidrug resistance in fecal carriers could lead to clinical infections and inflammatory diseases such as IBD [24, 25].

The rate of MDR isolates in ESBL-KP (82.6%) was much higher than that of ESBL-EC isolates. One of the possible reasons is that the simultaneous presence of three ESBL genes was significantly high in ESBL-KP. Also, the presence of *bla*_SHV_ which is predominant among the isolates of ESBL-KP is associated with resistance to different beta-lactam antibiotics [26]. On the other hand, the presence of virulent factors along with biofilm formation in ESBL-KP isolates was notable in the present study. The presence of MDR ESBL-KP isolates with the potency of high pathogenicity and type D biofilm formation could make its elimination difficult. In ESBL-KP which was isolated from fecal carriages, the presence of MDR isolates was 45.4%, which was lower than clinical isolates. However, MDR isolates were limited to fecal carriers in the ICU. On the other hand, the rate of some virulence factors, including *ompk35* and *ompk36* was as high as 86.3% and 77%, respectively (See also Table S1 in supplementary files). The presence of virulent ESBL-KP isolates with a high rate of biofilm formation (See also Figure S2 in supplementary files) that are resistant to different classes of antibiotics, specifically in the ICU, could be alarming. If these asymptomatic carriers become patients (due to the immunodeficiency that could occur in ICU patients), treatment could be more difficult.

Evaluation of MLVA typing methods in clinical samples revealed noticeable data. The results of MLVA in the present study revealed that some strains were in the same mini-cluster isolated from the same source and station. For instance, K9, K16, K41, K44, and K46 were isolated from tracheal samples in the ICU. K9 and K16 had the same MLVA profile and the same antibiotic resistance pattern and ESBL gene distribution were also observed in these two strainsIn addition to high molecular and phenotypic antimicrobial resistance, the presence of the *K2* geneassociated with liver abscesses, [27] with type D of biofilm formation in K16 could make this isolate difficult to eliminate and increase the risk of infection at other sites, including the liver. These data suggest that a ESBL-KP strain was circulatingin the ICU and if the infection control is performed appropriately, the circulation of infectious isolates will be limited. On the other hand, the spread of ESBL-KP isolates in different wards with the same source of infection was obsereved in some strains, including K34 and K36 isolated from joint samples from the emergency department and and the internal medicine department, respectively. This suggests that the same isolate could be transmitted in different settings and circulate among different patients.

Although MLVA analysis showed large heterogeneity between strains isolated from carriers and clinical samples, MLVA typing analysis between ESBL-KP, isolated from fecal caariers and clinical samples demonstrated significant data (See Fig. 1A, B, and also Figure S1 in supplementary files). As shown in Fig. 1B, some of the ESBL-KP isolated from fecal carriers and clinical samples were in the same mini-cluster. For instance, K49, K59, and K55 had a similar MLVA pattern. K49 and K59 were isolated from fecal carriages in the General ICU and K55 was isolated from a patient's joint sample from the Emergency ward. Evaluation of the molecular characteristics of these strains showed that all three strains were MDR and possessed similar virulence factor genes (see also Table S1 in supplementary files). Isolation of the ESBL-KP at different wards from the fecal carriage and clinical samples showed the possibility of high transmission of resistant isolates and colonization in asymptomatic carriers. On the other hand, these data might suggest that the presence of carriers could in turn, especially in hospital wards, increase the possibility of transmission to the hospitalized patient. Moreover, the current study also showed the presence of ESBL-KP isolates in carriers and clinical sources with a similar MLVA pattern in the same ward (K31 and K42). these data again demonstrated the probability of dissemination of ESBL-KP isolates between carriers and patients. The spread of the resistant isolates was also found between the community and the hospital. K50, the strain isolated from an outpatient fecal carriage, and K61 isolated from a tracheal sample from a hospital patient in the ICU, had a similar MLVA pattern. This suggests that ESBL-KP can be colonized with the same profiles in different settings and, therefore, may be widely distributed in both community and hospital settings.

## Conclusion

The presence of ESBL-KP isolates with a similar MLVA pattern in patients and asymptomatic fecal carriages could be alarming as it indicated the presence of hidden carriers. Also, the phylogroup B2 of ESBL-EC which is present in both clinical samples and fecal carriages is noteworthy in terms of carrier conversion to a pateint. Implementation of some control protocols, including monitoring of fecal carriages in the initial steps of hospital admission and also isolation of carriers in separate areas could impressively reduce the rate of fecal carriage and also the rate of infection.

### Supplementary Information

**Additional file 1: Table S1. **Clinical, phenotypic, and genotypic characteristics of the 22 ESBL-KP isolated from fecal carriage in Iran. **Table S2. **Antimicrobial resistance profile, Phylogroup typing, and ESBL genes distribution of 72 ESBL-EC isolated from fecal carriage in Iran. ** Figure S1. **Dendrogram based on MLVA of 22 ESBL-KP in fecal carriage isolates with a similarity cutoff of 80%.** Figure S2. **The rates of biofilm types in ESBL-KP isolated from fecal carriages. The yellow, blue, orange, and gray colors show the types of D, A, B, and C, respectively.

## Data Availability

The datasets generated during and/or analyzed during the current study are available from the corresponding author on reasonable request.

## Abbreviations

ESBL: Extended-spectrum beta-lactamase
ESBL-PE: ESBL producing *Enterobacteriaceae*
ICU: Intensive Care Unit
MLVA: Multiple locus variable-number tandem repeat analysis
CLSI: Clinical and laboratory standards institute
CAZ: Ceftazidime
CAZ/CLA: Ceftazidime / clavulanic acid
CTX: Cefotaxime
CTX/ CLA: Cefotaxime/ clavulanic acid
CPM: Cefepime
AK: Amikacin
GM: Gentamicin
CIP: Ciprofloxacin
LVX: Levofloxacin
ETP: Ertapenem
IMP: Imipenem
DDST: Double-disk synergy test
ESBL-KP: ESBL-producing *K. pneumoniae*
ESBL-EC: ESBL-producing *E. coli*
MDR: Multidrug resistance
OP: Outpatient
UC: Urine culture
BC: Blood culture
T/C: Tracheal culture
