# Supplementary material for: The notable relatedness between ESBL producing Enterobacteriaceae isolated from clinical samples and asymptomatic fecal carriers
Source: BMC Infect Dis. 2023 Nov 8;23:775. doi: 10.1186/s12879-023-08746-3 (PMC10634096; doi:10.1186/s12879-023-08746-3)
Supplement: Supplementary file 1 — Additional file 1: Table S1. Clinical, phenotypic, and genotypic characteristics of the 22 ESBL-KP isolated from fecal carriage in Iran. Table S2. Antimicrobial resistance profile, Phylogroup typing, and ESBL genes distribution of 72 ESBL-EC isolated from fecal carriage in Iran. Figure S1. Dendrogram based on MLVA of 22 ESBL-KP in fecal carriage isolates with a similarity cutoff of 80%. Figure S2. The rates of biofilm types in ESBL-KP isolated from fecal carriages. The yellow, blue, orange, and gray colors show the types of D, A, B, and C, respectively. [file 12879_2023_8746_MOESM1_ESM.docx]

**Table 1 S. Clinical, phenotypic, and genotypic characteristics of the 22 ESBL-KP isolated from fecal carriage in Iran**

| NO | unit | Resistance profile (disk diffusion) | ESBL genes | Virulence factor genes |
| --- | --- | --- | --- | --- |
| K67 | OP | CAZ,CTX | *bla_TEM_* | *ompk35,ompk36* |
| K5 | OP | CAZ,CTX | *bla_CTX-M15_* | - |
| K6 | OP | CAZ,CTX,CPM | *bla_CTX-M15_,bla_TEM_* | - |
| K64 | OP | CAZ,CTX,CPM,LVX,CIP | *Bla_CTX-M15_,bla_TEM_* | *ompk35* |
| K52 | E-ICU | CAZ,CTX,CPM,AK,GM,CIP,LVX,ETP,IMP | *bla_SHV_* | *ompk35* |
| K7 | E-ICU | CTX | *bla_CTX-M15_* | *ompk36* |
| K47 | OP | CAZ,CTX,CPM | *bla_CTX-M15_,bla_TEM_* | *fimH,mrkD,mrkA,fimA,ECP,KPN,entB,ompk35,ompk36* |
| K50 | OP | CAZ,CTX,CPM | *bla_CTX-M15_,bla_TEM_,bla_SHV_* | *fimH,mrkD,mrkA,fimA,ECP,KPN,entB,ompk35,ompk36* |
| K62 | G-ICU | CAZ,CTX | *bla_SHV_* | *fimH,mrkD,mrkA,KPN,entB,ompk35,ompk36,K2* |
| K13 | E-ICU | CTX | *bla_TEM_* | *fimH,mrkD,mrkA,KPN,entB,ompk35,ompk36* |
| K31 | E-ICU | CAZ,CTX,CPM | *bla_CTX-M15_,bla_SHV_* | *fimH,mrkD,mrkA,ECP,KPN,entB,ompk35,ompk36* |
| K39 | E-ICU | CAZ,CTX,CPM,AK,GM,CIP,LVX,ETP,IMP | *bla_CTX-M15_,bla_TEM,_bla_SHV_* | *fimH,mrkD,mrkA,KPN,entB,ompk35,ompk36* |
| K60 | G-ICU | CAZ,CTX,CPM,AK,GM,CIP,LVX,ETP,IMP | *bla_CTX-M15_,bla_TEM_,bla_SHV_* | *fimH,mrkD,mrkA,KPN,entB,ompk35,ompk36* |
| K63 | G-ICU | CAZ,CTX,CPM,AK,GM,CIP,LVX,ETP,IMP | *bla_CTX-M15_,bla_TEM_,bla_SHV_* | *fimH,mrkD,mrkA,ECP,KPN,entB,ompk35,ompk36* |
| K58 | G-ICU | CAZ,CTX,CPM,,LVX,CIP,IMP,ETP | *bla_CTX-M15_,bla_SHV_* | *fimH,mrkD,mrkA,fimA,ECP,KPN,entB,ompk35,ompk36* |
| K48 | G-ICU | CAZ,CTX,CPM | *bla_CTX-M15_,bla_TEM_,bla_SHV_* | *fimH,mrkD,mrkA,fimA,ECP,KPN,entB,ompk35,ompk36* |
| K65 | E-ICU | CAZ,CTX,CPM,AK,GM,CIP,LVX,ETP,IMP | *bla_CTX-M15_,bla_SHV_* | *fimH,mrkD,mrkA,fimA,KPN,entB,ompk35,ompk36* |
| K66 | E-ICU | CAZ,CTX,CFPM,LVX,CIP,AMK,GEN | *bla_CTX-M15_,bla_TEM_,bla_SHV_* | *fimH,mrkD,mrkA,fimA,KPN,entB,ompk35* |
| K68 | G-ICU | CAZ,CTX,CPM,AK,GM,CIP,LVX,ETP,IMP | *bla_CTX-M15_,bla_SHV_* | *fimH,mrkD,mrkA,fimA,entB,ompk35,ompk36* |
| K51 | G-ICU | CTX,CPM | *bla_CTX-M15_,bla_TEM_,bla_SHV_* | *fimH,mrkD,mrkA,fimA,ECP,KPN,entB,ompk35,ompk36,K2* |
| K49 | G-ICU | CAZ,CTX,CPM,AK,GM,CIP,LVX,ETP,IMP | *bla_CTX-M15_,bla_TEM_,bla_SHV_* | *fimH,mrkD,mrkA,ECP,KPN,entB,ompk35,ompk36* |
| K59 | G-ICU | CAZ,CTX,CPM,AK,GM,CIP,LVX,ETP,IMP | *bla_CTX-M15_,bla_TEM_,bla_SHV_* | *mrkD,mrkA,KPN,entB,ompk35,ompk36* |

G-ICU, general intensive care unit; E-ICU, emergency intensive care unit; OP, Outpatient; CAZ, ceftazidime; CTX, cefotaxime; CPM, cefepime; AK, amikacin; GM, gentamicin; CIP, ciprofloxacin; LVX, levofloxacine; ETP, ertapenem; IMP, imipenem

**Table 2 S. Antimicrobial resistance profile, Phylogroup typing, and ESBL genes distribution of 72 ESBL-EC isolated from fecal carriage in Iran.**

| NO. | Unit | Non susceptible profile | ESBL genes | Phylogroup |
| --- | --- | --- | --- | --- |
| E1 | OP | CAZ,CTX,CPM,CIP,LVX | *bla*_CTX-M15_*,bla*_TEM_ | B2 |
| E2 | OP | CAZ,CTX,CIP,LVX | *bla*_TEM,_*bla*_SHV_ | F |
| E3 | G-ICU | CAZ,CTX,CPM,CIP,LVX | *bla*_CTX-M15_ | F |
| E4 | E-ICU | CAZ,CTX,CPM,CIP,LVX | *bla*_TEM_ | F |
| E5 | OP | CAZ,CTX,CPM,CIP,LVX | *bla*_CTX-M15_*,bla*_TEM_ | B2 |
| E6 | G-ICU | CAZ,CTX,CIP,LVX,ETP | *bla*_CTX-M15_ | F |
| E7 | G-ICU | CAZ,CTX,CPM,GM,CIP,LVX | *bla*_CTX-M15_ | F |
| E8 | G-ICU | CAZ,CTX,CPM,CIP,LVX | *bla*_CTX-M15_ | D |
| E9 | G-ICU | CAZ,CTX,CPM,GM | *bla*_TEM_ | B2 |
| E10 | G-ICU | CAZ,CTX,CPM,CIP | *bla*_CTX-M15_ | B2 |
| E11 | E-ICU | CAZ,CTX,CPM | *bla*_CTX-M15_*,bla*_TEM_ | D |
| E12 | OP | CAZ,CTX,CPM | *bla*_CTX-M15_ | D |
| E13 | OP | CAZ,CTX,CPM | *bla*_CTX-M15_*,bla*_TEM_ | F |
| E14 | OP | CAZ,CTX,CPM | *bla*_CTX-M15_*,bla*_TEM_ | D |
| E15 | E-ICU | CAZ,CTX,CPM,CIP,LVX | *bla*_CTX-M15_ | D |
| E16 | OP | CTX | *bla*_CTX-M15X_*,bla*_TEM_ | B2 |
| E17 | OP | CAZ,CTX,CPM | *bla*_CTX-M15_*,bla*_TEM_ | B2 |
| E18 | OP | CAZ,CTX,CPM,GM,CIP,LVX | *bla*_CTX-M15_ | B2 |
| E19 | OP | CAZ,CTX,CPM,CIP,LVX | *bla*_CTX-M15_*,bla*_TEM_ | B2 |
| E20 | OP | CAZ,CTX,CPM | *bla*_CTX-M15_ | B2 |
| E21 | OP | CAZ,CTX,CPM,GM,CIP,LVX | *bla*_CTX-M15_ | B2 |
| E22 | OP | CAZ,CTX,CPM | *bla*_CTX-M15_*,bla*_TEM_ | B2 |
| E23 | OP | CAZ,CTX,CPM,CIP | *bla*_CTX-M15_ | B2 |
| E24 | OP | CAZ,CTX,CPM,CIP | *bla*_CTX-M15_*,bla*_TEM_ | B2 |
| E25 | G-ICU | CAZ,CTX,CPM,GM,CIP,LVX | *bla*_CTX-M15_ | B2 |
| E26 | E-ICU | CAZ,CTX,CPM,GM,CIP,LVX | *bla*_CTX-M15_ | B2 |
| E27 | OP | CAZ,CTX,CPM | *bla*_CTX-M15_ | D |
| E28 | OP | CAZ,CTX,CPM | *bla*_CTX-M15_ | D |
| E29 | OP | CAZ,CTX,CPM | *bla*_CTX-M15_*,bla*_TEM_ | D |
| E30 | OP | CAZ,CTX,CPM | *bla*_CTX-M15_*,bla*_TEM_ | D |
| E31 | G-ICU | CAZ,CTX,CPM | *bla*_CTX-M15_ | D |
| E32 | OP | CAZ,CTX | *bla*_SHV_ | F |
| E33 | OP | CAZ,CTX,CPM | *bla*_CTX-M15_ | F |
| E34 | G-ICU | CAZ,CTX,CPM | *bla*_CTX-M15_*,bla*_TEM_ | F |
| E35 | OP | CTX,CPM, CIP | *bla*_CTX-M15_ | A |
| E36 | OP | CAZ,CTX,CPM | *bla*_CTX-M15_*,bla*_TEM_ | A |
| E37 | OP | CAZ,CTX,CPM | *bla*_CTX-M15_*,bla*_TEM_ | A |
| E38 | OP | CAZ,CTX,CPM,CIP,LVX | *bla*_CTX-M15_ | A |
| E39 | OP | CTX,CPM | *bla*_CTX-M15_ | A |
| E40 | OP | CAZ,CTX,CPM | *bla*_TEM_ | A |
| E41 | OP | CAZ,CTX,CPM,CIP,LVX | *bla*_CTX-M15_ | A |
| E42 | OP | CAZ,CTX,CPM | *bla*_CTX-M15_*,bla*_TEM_ | A |
| E43 | OP | CAZ,CTX,CPM | *bla*_CTX-M15_*,bla*_TEM_ | A |
| E44 | OP | CAZ,CTX,CPM | *bla*_CTX-M15_ | A |
| E45 | G-ICU | CAZ,CTX,CPM | *bla*_CTX-M15_ | A |
| E46 | G-ICU | CAZ,CTX,CPM,CIP,LVX | *bla*_CTX-M15_ | A |
| E47 | G-ICU | CAZ,CTX,CPM | *bla*_CTX-M15_*,bla*_TEM_ | A |
| E48 | G-ICU | CAZ,CTX,CPM | *bla*_CTX-M15_ | A |
| E49 | E-ICU | CAZ,CTX,CPM | *bla*_CTX-M15_ | A |
| E50 | OP | CAZ,CTX,CPM | *bla*_CTX-M15_ | B1 |
| E51 | OP | CAZ,CTX | *bla*_CTX-M15_ | B1 |
| E52 | OP | CAZ,CTX,CPM | *bla*_CTX-M15_ | B1 |
| E53 | OP | CAZ,CTX,CPM | *bla*_CTX-M15_*,bla*_TEM_ | B1 |
| E54 | E-ICU | CAZ,CTX,CPM | *bla*_CTX-M15_ | B1 |
| E55 | G-ICU | CAZ,CTX,CPM,GM,CIP,LVX | *bla*_CTX_ | C |
| E56 | E-ICU | CAZ,CTX,CPM,CIP,LVX,ETP,IMP | *bla*_CTX-M15_*,bla*_TEM_ | C |
| E57 | OP | CTX,CPM | *bla*_CTX-M15_ | UT |
| E58 | OP | CTX,CPM | *bla*_CTX-M15_*,bla*_TEM_ | UT |
| E59 | OP | CAZ,CTX,CPM | *bla*_CTX-M15_ | UT |
| E60 | OP | CAZ,CTX,CPM | *bla*_CTX-M15_*,bla*_TEM_ | UT |
| E61 | OP | CAZ,CTX,CPM,CIP,LVX | *bla*_CTX-M15_*,bla*_TEM_ | UT |
| E62 | G-ICU | CTX,CPM,CIP | *bla*_TEM_ | UT |
| E63 | G-ICU | CAZ,CTX,CPM,GM,CIP,LVX | *bla*_CTX-M15_*,bla*_TEM_ | UT |
| E64 | G-ICU | CTX,CPM,CIP,LVX | *bla*_CTX-M15_*,bla*_TEM_ | UT |
| E65 | G-ICU | CAZ,CTX,CPM | *bla*_CTX-M15,_*bla*_SHV_ | UT |
| E66 | G-ICU | CAZ,CTX,CPM,GM | *bla*_CTX-M15_*,bla*_TEM_ | UT |
| E67 | G-ICU | CAZ,CTX,CPM,CIP,LVX | *bla*_CTX-M15_*,bla*_TEM_ | UT |
| E68 | G-ICU | CAZ,CTX,CPM | *bla*_TEM_ | UT |
| E69 | G-ICU | CAZ,CTX,CPM,AK,GM,CIP,LVX,ETP,IMP | *bla*_CTX-M15,_*bla*_SHV_ | UT |
| E70 | E-ICU | CAZ,CTX,CPM,GM,CIP,LVX | *bla*_CTX-M15_*,bla*_TEM_ | UT |
| E71 | E-ICU | CAZ,CTX,CPM,CIP,LVX | *bla*_CTX-M15_*,bla*_TEM_ | UT |
| E72 | E-ICU | CAZ,CTX,CPM | *bla*_CTX-M15_*,bla*_TEM_ | UT |

G-ICU, general intensive care unit; E-ICU, emergency intensive care unit; OP, Outpatient; CAZ, ceftazidime; CTX, cefotaxime; CPM, cefepime; AK, amikacin; GM, gentamicin; CIP, ciprofloxacin; LVX, levofloxacine; ETP, ertapenem; IMP, imipenem


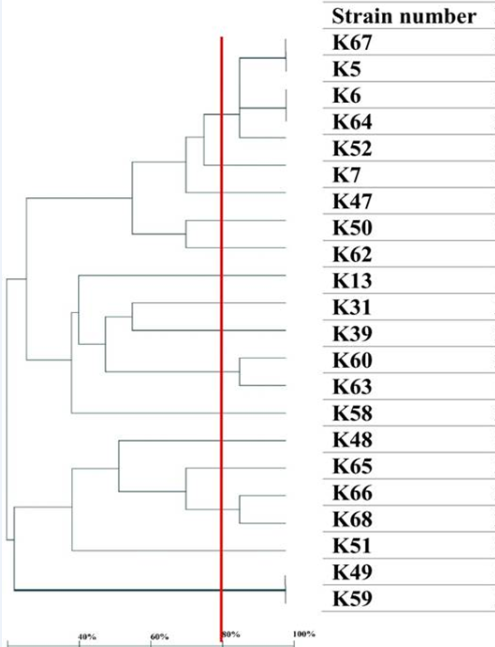


**Figure 1 S.** Dendrogram based on MLVA of 22 ESBL-KP in fecal carriage isolates with a similarity cutoff of 80%.

**Figure 2 S.** The rates of biofilm types in ESBL-KP isolated from fecal carriages. The yellow, blue, orange, and gray colors show the types of D, A, B, and C, respectively.
